# Dietary equity for Indian migrant women in Auckland: why culturally grounded evidence is urgently needed

**DOI:** 10.1017/S1368980026103000

**Published:** 2026-06-24

**Authors:** Laltlankimi Khiangte

**Affiliations:** School of Community and Public Health, Auckland University of Technology, New Zealand

**Keywords:** Dietary equity, Public health nutrition, Indian migrant women, Cultural relevance, Public policy

## Abstract

Most Indian communities form a significant and growing component of the Auckland population, including Indian migrant women, who often play central roles in food procurement and household dietary practices. While New Zealand and international evidence indicates post-migration dietary changes and barriers to healthy eating among South Asian populations, there is limited Auckland-specific information on the dietary behaviour of Indian migrant women. This commentary integrates existing evidence and outlines high-level research, policy and practice priorities. Strengthening culturally grounded dietary assessment, while addressing multilevel factors such as affordability, food environments and access to culturally appropriate information, is essential for promoting nutrition equity among the largest Asian ethnic female populations living in Aotearoa New Zealand.

The Indian population is growing in New Zealand. According to Stats NZ Aotearoa Data Explorer (2025), the national ethnic population projection shows the Indian population is likely to grow significantly between 2023 and 2048; this growth is expected to be approximately 130 % from 2023 (338 000 people) to 2048 (772 500 people)^(1)^. This trend can be seen in Auckland, New Zealand, where Indian people are the second-largest Asian group, making up 35·4 % of the Asian ethnic groups living in Auckland^(2)^. In Indian families, women usually play the main role in buying and preparing food, and as a result, they also influence the family’s eating habits and health outcomes^(3–5)^. However, research is scarce on Auckland’s Indian migrant women and their dietary behaviour, which is extremely puzzling considering the integral role diet plays in the prevention of chronic diseases, which are alarmingly prevalent in South Asian communities^(6,7)^.

## Post-migration dietary change

South Asian migrants, such as Indian migrants, when migrating to high-income countries such as New Zealand, Australia, Canada, the United Kingdom, and the United States, have repeatedly demonstrated changes in diet, including greater exposure to ultra-processed foods, less consumption of traditional staples and shifts prompted by affordability, availability and time scarcity^(8,9)^. In New Zealand, South Asian migrants are less likely to consume fruits, vegetables, legumes, whole grains and dairy/calcium in accordance with the dietary guidelines for New Zealand, while the consumption of takeaway foods and junk foods has increased^(6,7)^. Previous studies conducted in Auckland have confirmed ethnic differences in calcium intake, and these may have implications for the bone health of Asian women^(10)^.

None of these changes happens alone. They are influenced by a tangled combination of cultural choices, foodscapes, gender responsibilities and socio-economic limitations^(11)^. Convenience foods can be a substitute for traditional dishes among many migrants, often as a result of long working hours and lack of access to cultural ingredients or the time required for labour-intensive cooking^(8)^.

## Cross-cultural applicability of dietary assessment instruments

In this context, the nutritional assessment instruments developed for the general population may not represent the full range and complexity of the Indian migrant diet in New Zealand. Previous New Zealand work suggests that composite dishes, pulse-based dishes and culturally specific whole-grain products may be under-recorded in generic tools^(6,7)^. This can result in an underestimation of diet quality and in the inability to compare migrant diets with national recommendations.

Meaningful improvements in the cultural validity of dietary assessment are needed, however, because accurate research is only one-half of why this is important; dietary guidance must also be relevant and realistic for Indian populations. Lessons from South Asian co-design projects might provide valuable guidance in this regard^(12,13)^, particularly in understanding how broader food system factors shape dietary behaviour.

## Facilitators and barriers across the food system

Dietary behaviour does not occur in isolation; it is influenced by larger ecosystem elements, including interpersonal, organisational, community and environmental factors^(14)^. Migrant populations’ diets are affected by a variety of structural, environmental and cultural factors that can impede access to healthy food^(8,9)^. The following represent some of the significant impediments located at various levels of the food system that may affect the dietary behaviours of Indian migrant women in Auckland, New Zealand:

- Affordability

Food-price inflation and increasing prices for nutrient-rich foods, especially dairy, have the potential to push households toward cheaper discretionary items^(15)^. National evaluations have demonstrated that sweetened or ultra-processed food products are now relatively cheaper than fresh produce^(16)^. Market structure characteristics, such as the effects of supermarket duopoly, also affect affordability^(15,17)^.

- Food environments

Availability of culturally specific foods within Auckland also varies from neighbourhood to neighbourhood, and in some cases, residents may need to seek out food at speciality ethnic outlets^(18)^.

- Navigation and trust

Asian immigrants frequently report difficulties accessing primary care, such as poor language assistance and an unfamiliar health system^(19)^. These obstacles limit the availability of preventive nutritional counselling.

- Social and cultural context

Because of cross-cultural expectations, stereotypical roles and shameful feelings may affect help-seeking and responses to dietary information among Indian females^(3)^. These combined multilevel effects indicate that intervention strategies aimed at modifying diet must move beyond individual-level knowledge to tackle broader structural and environmental determinants.

## Why Auckland-specific evidence is required

Despite Indians representing one of the largest migrant populations in Auckland^(2)^ and growing recognition of the food system barriers and facilitators influencing the dietary behaviour of migrants, important evidence gaps remain regarding Indian migrant women. Pregnancy-based studies have contributed significantly to our understanding of dietary compliance among women resident in New Zealand^(20,21)^. However, limited research has examined the dietary behaviours and the contextual drivers shaping these behaviours among Indian migrant women who became the largest Asian migrant ethnic female group in New Zealand since 2023 (157 000 females)^(1)^. Stats NZ Tatauranga Aotearoa further estimates that the Indian migrant female population will increase to 368 600 by 2048^(1)^.

This gap hinders the design of nutrition interventions for local regions. Evidence from South Asian health-promotion and co-design approaches suggests that culturally adapted^(6,7)^ strategies hold promise but rely upon accurate population-specific data.

## An ambitious research and action agenda

To improve the effectiveness of nutrition policies and practices for migrant populations, such as Indian migrants in Auckland, it is important to understand how the dietary behaviours of Indian women are shaped within the migrant context. Once this has been accomplished, a coordinated research and action plan can be developed to focus efforts on supporting cultural identity, local diets, social/physical determinants of health and community-based food system solutions that make diets consistent with public health recommendations more accessible. Key areas for consideration include the following:

1. Enhancing the cultural relevance of diet assessments: To effectively assess, tools must also be designed to represent the practices in Indian food habits, such as mixed dishes, pulse-based staples, culinary methods used and whole-grain diversities.
2. Developing Auckland-specific dietary profiles: Indian women’s dietary behaviours in the context of the city, demography and food-environment are involved and require focused research.
3. Considering multilevel determinants: Future work should consider affordability, access, language and navigation barriers, as well as food-environment constraints.
4. Supporting culturally responsive nutrition services: There may be potential benefits to interpreter support, culturally relevant examples and partnerships with the community to help build trust and access.
5. Collaborative food system solutions: Involvement of supermarkets, ethnic retailers, local health agencies and community partners can help enhance affordability and access to guideline-aligned foods.

## Conclusion

Indian migrant women are one of the largest migrant groups in Auckland, New Zealand, and have a strong influence on household dietary practices, but studies have not investigated the dietary behaviour of this population. Available evidence indicates gaps in core food groups and barriers reflecting affordability, access and navigation. These gaps will need to be addressed with culturally appropriate measures, Auckland-specific data and multilevel interventions. It is important to build on such evidence, which should be used to inform the development of effective and equitable nutrition services for one of New Zealand’s rapidly increasing population groups.
